# Polymorphous corneal dystrophy subtype 3 and keratoconus aggravation after corneal refractive surgery in a three-generation family carrying both *ZEB1* and *ZNF469* pathogenic variant

**DOI:** 10.3389/fgene.2025.1603019

**Published:** 2025-06-06

**Authors:** Qinghong Lin, Xuejun Wang, Xiaoliao Peng, Xiaosong Han, Xiaoyu Zhang, Ling Sun, Yan Wang, Shengtao Liu, Xingtao Zhou

**Affiliations:** ^1^ Department of Ophthalmology, Eye and ENT Hospital of Fudan University, Shanghai, China; ^2^ Eye Institute and Department of Ophthalmology, Eye and ENT Hospital, Fudan University, Shanghai, China; ^3^ NHC Key Laboratory of Myopia (Fudan University), Key Laboratory of Myopia, Chinese Academy of Medical Sciences, Shanghai, China; ^4^ Shanghai Research Center of Ophthalmology and Optometry, Shanghai, China; ^5^ Shanghai Engineering Research Center of Laser and Autostereoscopic 3D for Vision Care (20DZ2255000), Shanghai, China; ^6^ Refractive Surgery Department, Bright Eye Hospital, Fuzhou, China

**Keywords:** keratoconus (KC), ZNF469, Zeb1, posterior polymorphous corneal dystrophy (PPCD), corneal refractive surgery

## Abstract

**Background:**

This study reports a three-generation Chinese family with polymorphous corneal dystrophy subtype 3 (PPCD3) and keratoconus (KC) aggravation induced by corneal refractive surgery, specifically small incision lenticule extraction (SMILE), in the context of genetic variations.

**Methods:**

The history of illnesses and blood samples of all family members were collected. One hundred healthy individuals served as normal controls. We conducted whole exome sequencing on genomic DNA and sanger sequencing to verify the variants between all controls and family members.

**Results:**

Three family members were previously diagnosed with subclinical keratoconus (III1 and III2 preoperatively, and II2). Both the proband (III1) and her younger brother (III2) underwent SMILE to correct refractive errors. One year later, visual acuity of III1 decreased significantly with KC aggravation and corneal opacification. The KC of III2 progressed significantly 6 months after surgery. Both were subsequently diagnosed with PPCD3. We detected both Zinc finger E-box-binding homeobox 1 (ZEB1) gene and zinc finger protein 469 (ZNF469) gene pathogenic variant in the proband and another two patients in this family, including a heterozygous missense variation c.13C>G (p.P5A, rs753301298) in the *ZEB1* gene, and a heterozygous non-frameshift variant c. 3093_3104del (p.D1035_K1038del) in the *ZNF469* gene. The variants including c.13C>G in *ZEB1* and c.3093_3104del in *ZNF469* were speculated to be pathogenic or a variant of uncertain significance by online prediction software.

**Conclusion:**

This study demonstrated the importance of a thorough ocular examination, especially the cornea, and a gene screening before SMILE.

## 1 Introduction

Corneal refractive surgery includes various modalities, including laser-assisted *in situ* keratomileusis (LASIK), photorefractive keratectomy (PRK), and small-incision lenticule extraction (SMILE). Recent studies have suggested that post-refractive keratoconus (KC) occurs from the lowest to highest rate in eyes that have undergone SMILE, PRK and LASIK, respectively. However, given the increasing number of people who have undergone SMILE, post-SMILE keratoconus is a growing concern. Since keratoconus is a spectrum of disease, pre-existing keratoconus is more important in postoperative ectasia than previously thought (Moshirfar et al., 2021; Zhang et al., 2022).

Keratoconus is a progressive disease characterized by corneal thinning, lack of inflammation, irregular curvature, and scar formation, which can lead to severe visual loss in later stages and is often accompanied with other systemic and/or eye diseases (Bykhovskaya et al., 2016; Loukovitis et al., 2018). Variations of *ZEB1* and *ZNF469*, which are identified in corneal dystrophy, have been suggested to be related to keratoconus (Karolak et al., 2020; Zhang et al., 2021). In addition, environmental factors also play important roles (Crawford et al., 2020).

Posterior polymorphous corneal dystrophy (PPCD) and keratoconus have been co-occurrent in many patients (Burdon and Vincent, 2013; Fernández-Gutiérrez et al., 2022). In this study, a three generation Chinese family with keratoconus and PPCD subtype 3 (PPCD3) was studied. A total of three members in the family were observed to have keratoconus and PPCD3, among which two members (including the proband) had undergone SMILE to correct refractive errors. Keratoconus is a progressive corneal disorder that may be associated with genetic factors, and new pathogenetic variants of ZEB1 and ZNF469 were identified in this study. Additionally, PPCD3 can further complicate the clinical course of KC, especially after surgical interventions. The increasing understanding of the genetic underpinnings of these conditions highlights the importance of early diagnosis and gene screening, particularly for patients who may be at risk of postoperative complications like keratectasia or the progression of KC.

## 2 Materials and methods

### 2.1 Participants and examinations

There were 105 participants in this study, including five living family members from a three-generation Chinese family with PPCD3 and KC, and 100 unrelated healthy Chinese individuals, who were not diagnosed with PPCD, KC or other inherited corneal disorders. In the family, two members, including the proband (III1) and her younger brother (III2), underwent SMILE to correct refractive errors. All family members denied allergies, habitual eye rubbing, and trauma.

All participants provided written informed consent, and underwent detailed ophthalmic (i.e., best-corrected visual acuity (BCVA), biomicroscopy, and fundus examination) and physical examinations. In addition, the Scheimpflug camera system (Pentacam; Oculus Optikgeräte GmbH, Wetzlar, Germany) and optical coherence tomography (OCT), (Heidelberg Spectralis Heidelberg Engineering Gmbh, Germany) were used for corneal examination. The corneal endothelium cell density (ECD) was measured by non-contact specular microscopy (SP-2000P, Topcon Corporation, Japan). This study was approved by the Institutional Review Board of Fudan University (Shanghai, China) (approval no. 2022128) and was performed in compliance with the Declaration of Helsinki.

### 2.2 Whole exome sequencing

Exome sequencing (ES) was performed for 3 participants (III:1, III:2 and II:2) using the method previously described (Lin et al., 2022). Genomic DNA was extracted from leukocytes, and the exonic sequences were enriched, after which data processing and analyses were conducted. The 1000 Genomes Project was used to examine reported variants and those presented in patients with corneal dystrophy or KC at frequencies ≤1%. Only the variants shared in affected family members, namely III:1, III:2 and II:2 were considered as candidate variants.

### 2.3 Variant validation and analysis

Variant validation and analyses were performed. All variations were analyzed using online software, including Polyphen2 (genetics.bwh.harvard.edu), SIFT (sift.jcvi.org), fathmm-MKL (http://fathmm.biocompute.org.uk), CADD v1.4 (cadd.gs.washington.edu), Variant Taster (varianttaster.org) and ACMG guidelines (American College of Medical Genetics and Genomics). Subsequently, candidate variants were confirmed using polymerase chain reaction (PCR) and Sanger sequencing. The PCR primers were designed using Primer3. The validation and analyses were conducted according to the NCBI VARIANT (https://www.ncbi.nlm.nih.gov/clinvar/), NCBI HomoloGene (https://www.ncbi.nlm.nih.gov/guide/howto/find-homolog-gene/), and 1000 Genomes Project (https://www.internationalgenome.org/) databases. Three-dimensional (3D) protein structures of the variants were generated using the online server I-TASSER (https://zhanggroup.org/I-TASSER/).

### 2.4 Analysis of the protein-protein interaction network

Search Tool for the Retrieval Interacting Genes 11.5 (STRING) (https://cn.string-db.org) was used for online analysis of the protein-protein interaction network (PPI), which was then imported into Cytoscape (v3.9.0). Degree ≥5 was set to select significant proteins among the networks.

## 3 Results

### 3.1 Clinical manifestations

**FIGURE 1:**
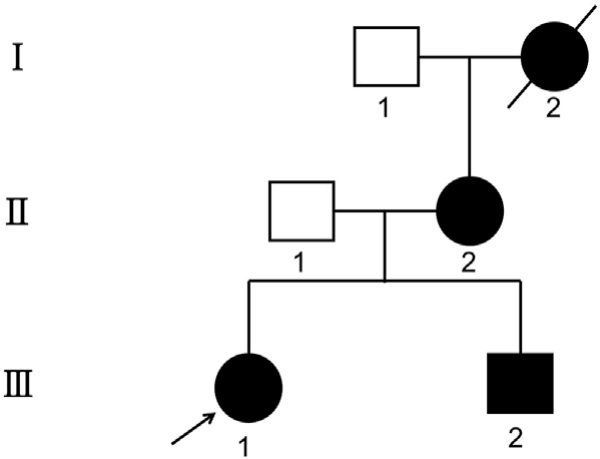
The genogram of a three-generation Chinese family affected by posterior polymorphous corneal dystrophy (PPCD3) and keratoconus (KC). The squares represent male members, while circles indicate female members. The solid symbols show that the individual has both PPCD3 and KC. The open symbols represent the unaffected family members. Subject III.1 was the proband (arrow).

The pedigree is shown in Figure 1, and the corresponding clinical data are summarized in Table 1. In this family, three living members developed KC and PPCD3, among which III:2 and III:1 were diagnosed with subclinical KC preoperatively and underwent SMILE surgery in October 2021. One year later, the visual acuity of the proband (III:1) declined significantly due to keratoconus aggravation. Her uncorrected distance visual acuities (UDVAs) were 10/50 in both eyes, corrected distance visual acuities (CDVAs) were 30/50 (right eye) with −1.00DS/-1.50 DC × 175° correction and 30/50 (left eye) with −1.50 DS/-1.50 DC × 15° correction (Supplementary Figure S1). For subject III:2, keratoconus aggravated significantly 6 months after SMILE; the UDVAs were 20/50 in both eyes, and the CDVAs were 40/50 (right eye) with −1.00DS/-1.00 DC × 170° correction and 30/50 (left eye) with −2.00 DS/-2.00 DC × 175° correction. A postoperative evaluation found that the maximum anterior surface curvature (MASC) and posterior elevation of the cornea (PEC) at the thinnest point of the cornea significantly increased, indicating that the keratoconus had worsened (Supplementary Figure S2). Moreover, these values showed a continuous upward trend in the subsequent follow-up.

**TABLE 1:** The data of family members.

Family member	Gender/Age	SMILE surgery	Diagnosis	PEC (µm)			CDVAs	Genetic findings
				Pre-SMILE	6 m post SMILE	1y post SMILE		
III:1	Female/37	Yes Bilateral	Keratoconus PPCD3	9(OD) 14(OS)	6(OD) 29(OS)	19(OD) 44(OS)	−1.00DS/-1.50 DC × 175°→30/50 (OD), −1.50 DS/-1.50 DC × 15°→30/50 (OS)	ZNF469(p.D1035_K1038del) ZEB1 (p.P5A)
III:2	Male /35	Yes Bilateral	Keratoconus PPCD3	14(OD) 13(OS)	26(OD) 30(OS)		−1.00DS/-1.00 DC × 170°→40/50 (OD), −2.00 DS/-2.00 DC × 175°→30/50 (OS)	ZNF469(p.D1035_K1038del) ZEB1 (p.P5A)
II:1	Male /67	No	—	7(OD) 7(OS)	—	—	—	—
II:2	Female/60	No	subclinical keratoconus PPCD3	18(OD) 14(OS)	—	—	−1.00DS→20/20 (OD), −1.50 DS→20/20 (OS)	ZNF469(p.D1035_K1038del) ZEB1 (p.P5A)
I:1	Male/80	No	IOL postoperative	—	—	—	—	—
I:2	Female/78	Deceased		—	—	—	—	—

**FIGURE 2:**
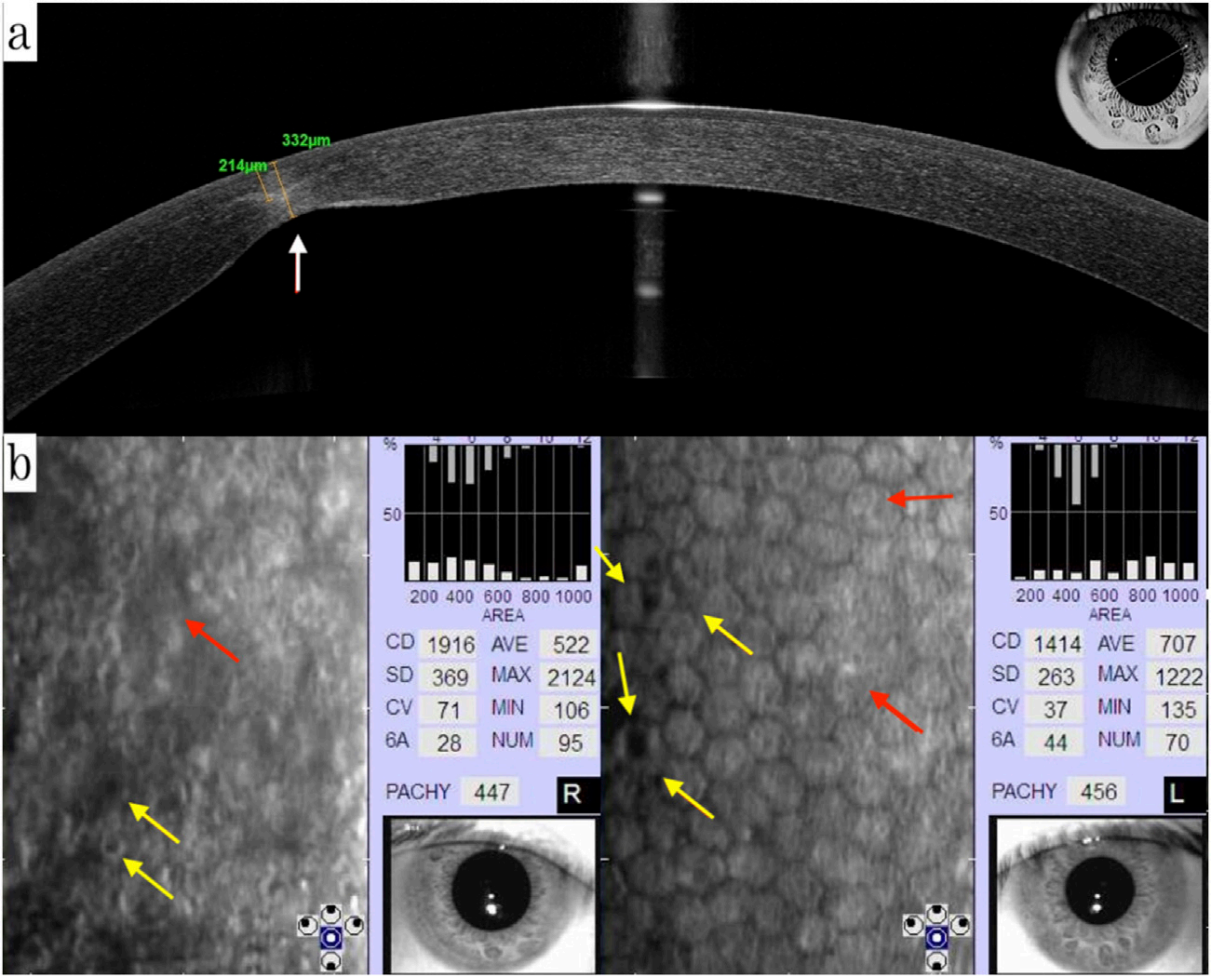
**(a)** OCT showing the corneal endothelial and stromal opacification of the left eye (white arrow); **(b)** ECD of the proband was reduced significantly to only 1914 cells/mm2 in the right eye and 1414 cells/mm2 in the left eye. Polymorphous, giant endothelial cells with some nucleated cells (red arrow), and hypo-reflective vesicular lesions, in the form of a crater with hyperreflective deposits around the lesions, were shown in both eyes (yellow arrow). OCT: optical coherence tomography, ECD: endothelium cell density.

Greyish opacification of the corneal endothelium and stroma in the subnasal region and endothelial rail tracks were observed in the left eye by biomicroscopy and OCT (Figures 2a,b). OCT showed the corneal endothelial and stromal opacification of the left eye. Moreover, ECD of the proband reduced significantly to only 1914 cells/mm^2^ in the right eye and 1414 cells/mm^2^ left eye. Polymorphous, giant endothelial cells with some nucleated cells and hypo-reflective vesicular lesions, in the form of a crater with hyperreflective deposits around the lesions, were shown in both eyes. which is consistent with phenotypes of PPCD3. Subject II:2, the mother of the proband, was also diagnosed with subclinical keratoconus through a pentacam examination. The rough image displayed abnormal increase of the posterior corneal surface. (Supplementary Figure S3).

### 3.2 Identification and analysis of the new variants

**FIGURE 3:**
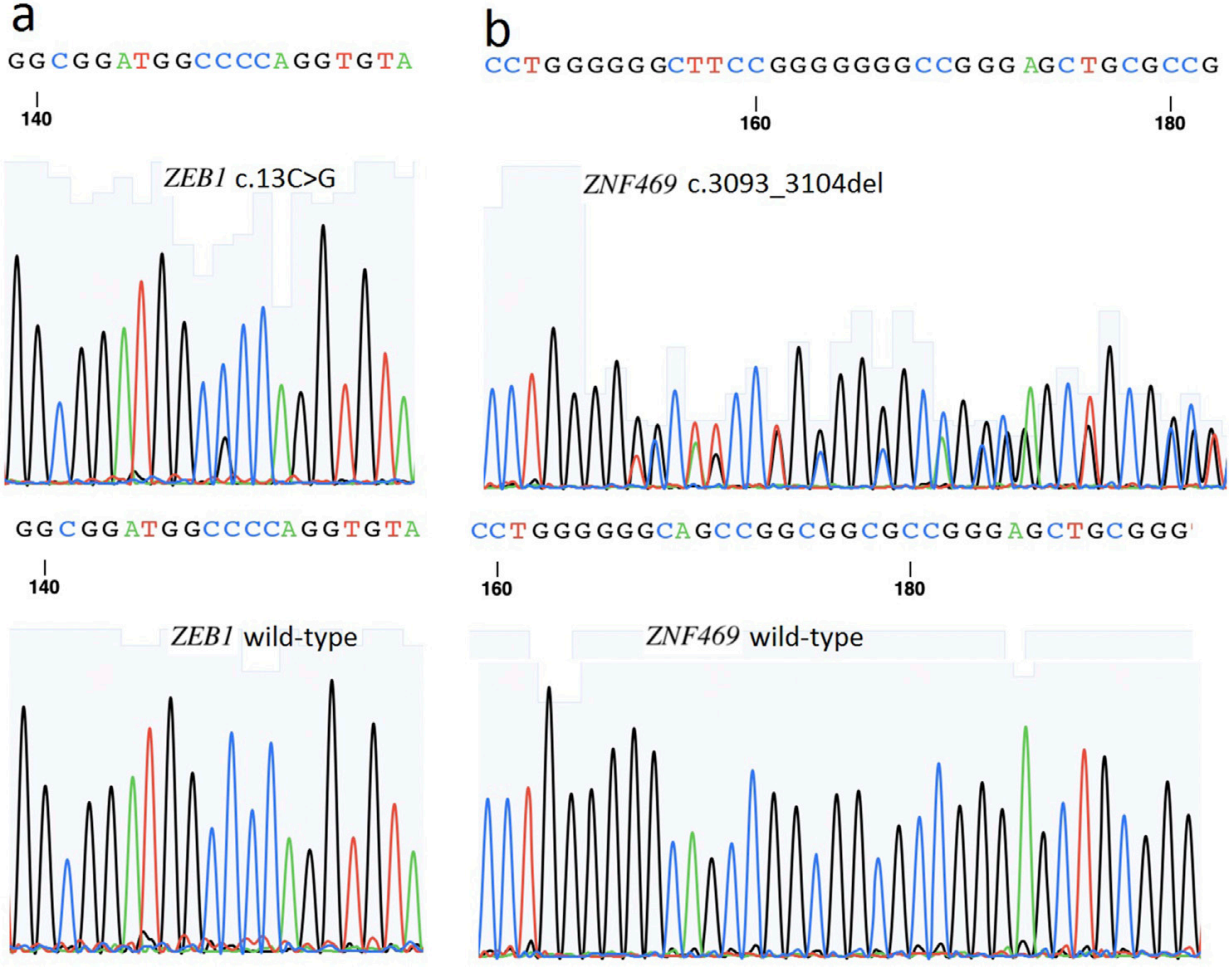
**(a)** A heterozygous non-frameshift variant c. 3093_3104del (p.D1035_K1038del) in *ZNF469* gene is shown (arrow). **(b)** A heterozygous missense variant c. 13C>G (p.P5A, rs753301298) in *ZEB1* gene is shown (arrow).

Two new variants were identified in this three-generation family: a heterozygous non-frameshift variant c. 3093_3104del (p.D1035_K1038del) in the *ZNF469* gene, and a heterozygous missense variation c.13C>G (p.P5A, rs753301298) in the *ZEB1* gene (Figures 3a,b). Variant c.3093_3104del (delCCCCAGGAAGGA) in *ZNF469* is in exon 3, which leads to a four amino acid deletion from 1035 to 1038 in the zinc finger protein. p.P5A in *ZEB1* is in exon 1, the amino-terminus (NZF) of the zinc finger E-box-binding homeobox 1 protein. These two variations were detected in III:2, III:1 and II:2. The other two healthy members in this family did not carry these variations. Family member I:2 had passed away 2 years prior to this study but had experienced corneal opacity resulting in blindness prior to her death. All variants were absent in the 100 random controls.

**FIGURE 4:**
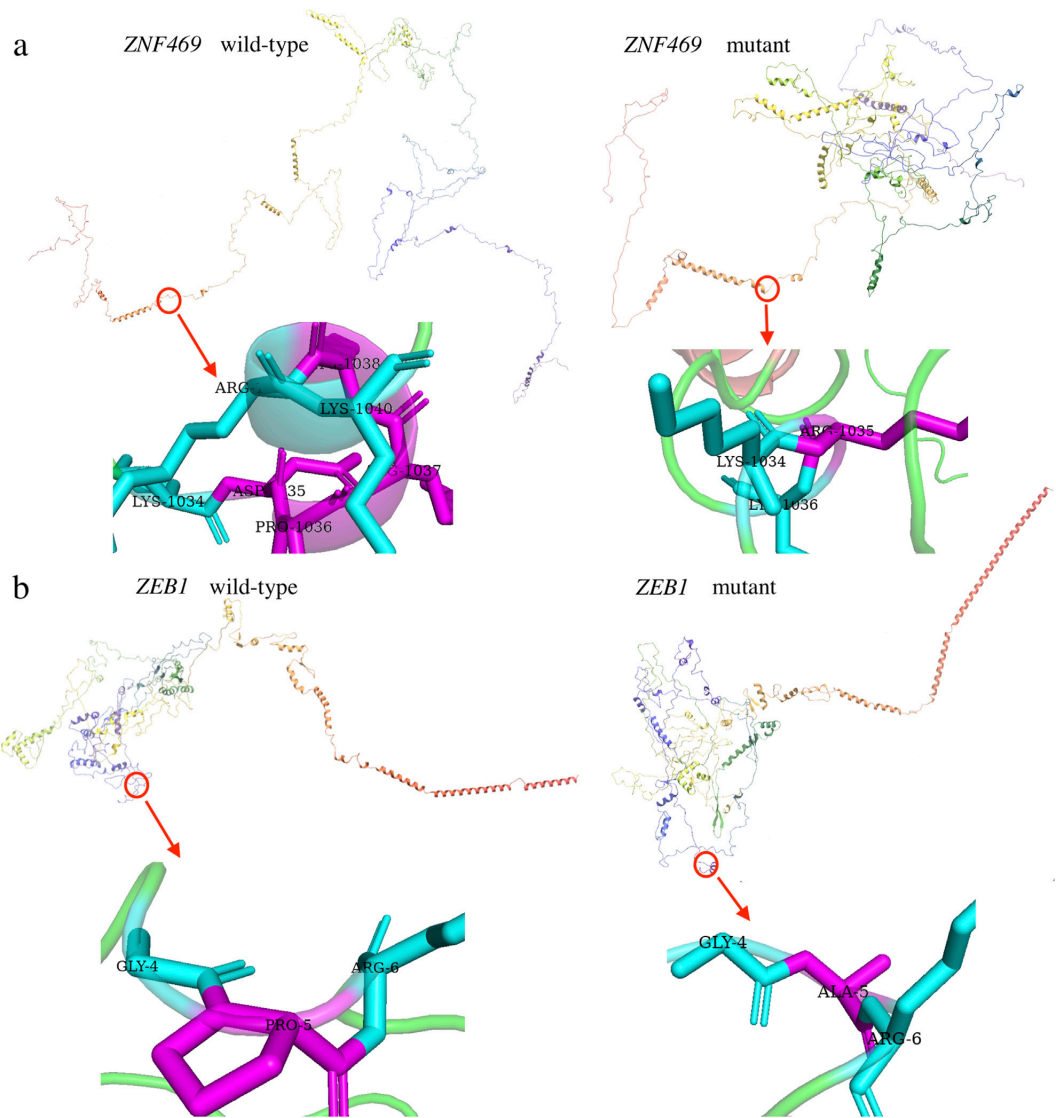
Three dimensional (3D) models of the proteins highlighting the variant sites. The inset pictures show the variants. **(a)** 3D modeling of wild-type *ZNF469* and p.D1035_K1038del variant. **(b)** 3D modeling of wild-type *ZEB1* and p.P5A variant.

The variant c. 3093_3104del (p.D1035_K1038del) in *ZNF469* was predicted to be a “Polymorphism” by Varianttaster Prediction, however, this variation resulted in the deletion of four amino acids from 1035 to 1038. According to the ACMG guidelines, the variant was predicted to be variant of uncertain significance (VUS), for it had not been reported in Human Gene Variant Database (HGMD) (PM2) and the change in protein length was the result of an in-frame deletion (PM4). The 3D modeling of the wild-type protein and the variation clearly displayed the conformational changes induced by the variant (Figure 4a). A single nucleotide polymorphism (SNPs) c.13C>G (p.P5A, rs753301298) in *ZEB1* gene was suggested to be pathogenic by online prediction programs, including Polyphen2, SIFT, VariantTaster and Fathmm-MKL. The CADD (Combined Annotation Dependent Depletion) score is 25.1, indicating “Probably Deleterious” (range from 25.0 to 29.9) (Table 2). Moreover, conformational changes related to the variant were exhibited by the 3D modeling of *ZEB1* wild-type and its variant (Figure 4b). The allele frequency of the SNP rs753301298 in the normal population was 0.00001 (database: gnomAD_exome_EAS), which is very rare.

**TABLE 2:** Identification and analysis of the new variants.

Gene	Variant	Phenotype	rs number	Polyphen2 prediction	SIFT prediction	Variant taster prediction	Fathmm-MKL	CADD
*ZNF469*	c.3093_3104delCCCCAGGAAGGA	Nonframeshift Deletion (p.D1035_K1038del)	—	—	—	Polymorphism	—	VUS
*ZEB1*	c.13C>G	Nonsynonymous (p.P5A)	rs753301298	Probably damaging	Deleterious	Disease causing	Deleterious	Probably Deleterious

### 3.3 Analysis of the protein-protein interaction network

**FIGURE 5:**
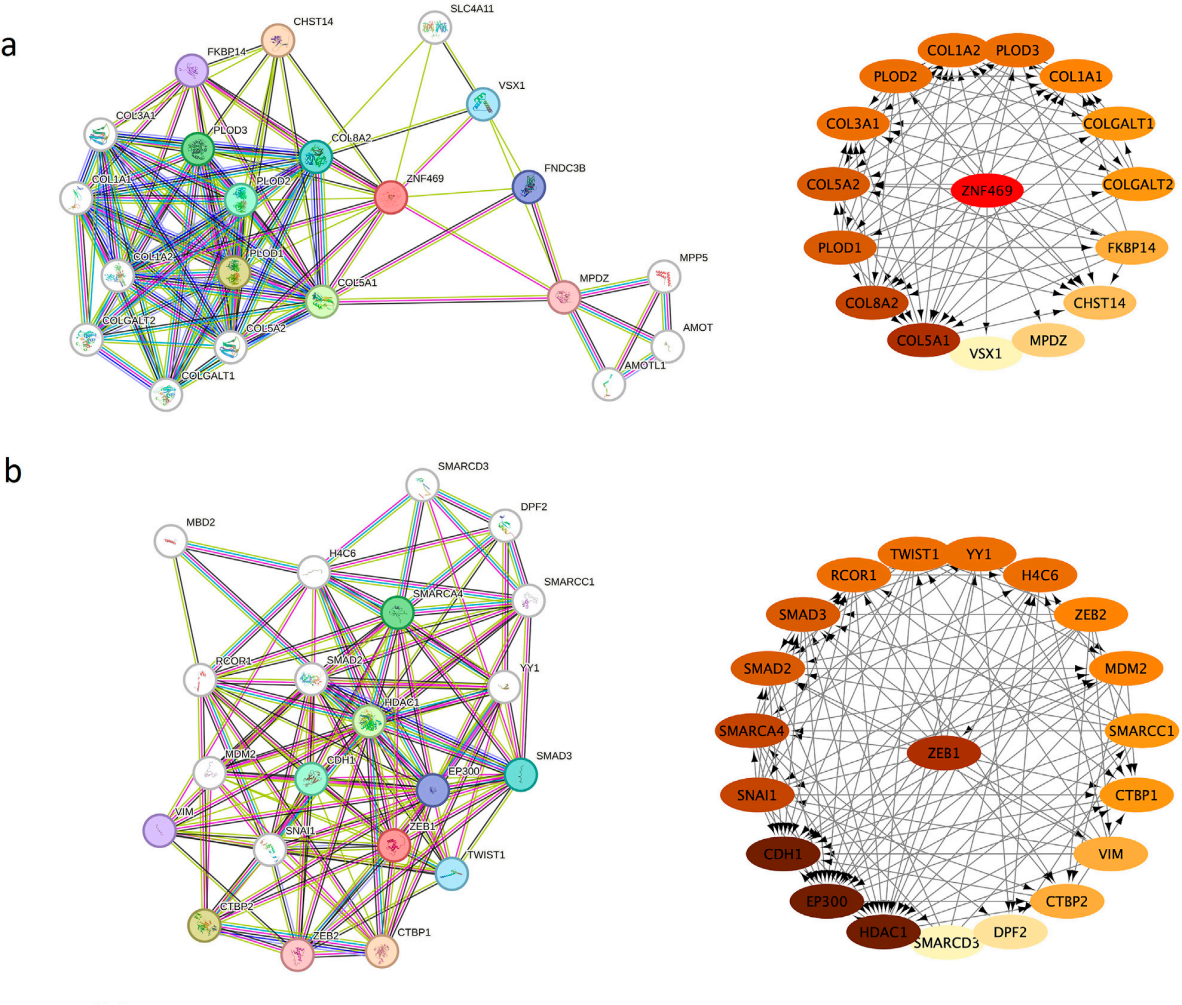
**(a)** The protein interaction network constructed by STRING and the network of PPI network performed using Cytoscape for ZNF469; **(b)** The protein interaction network constructed by STRING and the network of PPI network performed using Cytoscape for ZEB1.

Sixteen and twenty significantly enriched genes were uploaded to STRING to generate the PPI network for *ZNF469* and *ZEB1*, respectively, and the results were subsequently imported to Cytoscape to construct sub-networks. In the network, *ZNF469* and *ZEB1* are placed and highlighted in the middle (*ZNF469* proteins with the high degree of 13, close to COL5A1 and COL8A2, and *ZEB1* proteins with the degree of 15.0, which is next to HDAC1, EP300 and CDH1) (Figures 5a,b).

## 4 Discussion

### 4.1 Pathogenic variants in ZEB1 and ZNF469 are significantly associated with both PPCD3 and KC

Posterior polymorphous corneal dystrophy (PPCD) is a rare autosomal dominant disorder with genetic heterogeneity, primarily characterized by corneal endothelial abnormalities. These defects manifest as opacities and distinct lesions, including Descemet’s membrane bands and endothelial cell vesicles in the posterior corneal layers (Weiss et al., 2015; Lin et al., 2016). Currently, three genetically distinct subtypes have been identified: The gene(s) responsible for the PPCD1 subtype is *OVOL2*. PPCD3 is due to mutations in *ZEB1*. PPCD4 is linked to the gene *GRHL2*, and accounts for about 30% of individuals with PPCD and often is associated with corneal steepening (Davidson et al., 2020).

**FIGURE 6:**
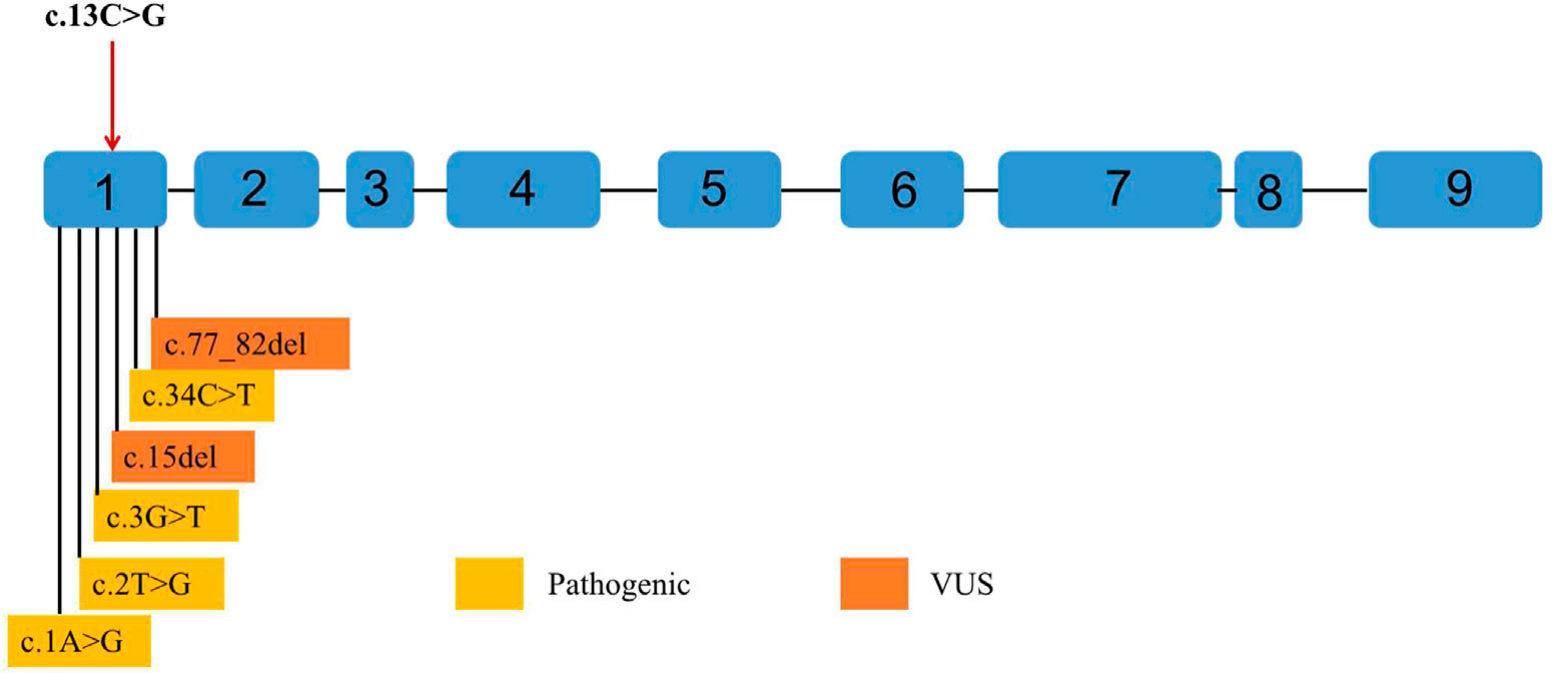
Point variants classified as pathogenic and variants of uncertain significance (VUS) by the American College of Medical Genetics and Genomics (ACMG) observed in *ZEB1* exons up to June 2023. Variants classified as pathogenic are shown in yellow. Variants classified as VUS are shown in orange. Any point variant classified as “likely pathogenic” has been reported in the Leiden Open Variation Database (LOVD). The reference transcript employed is NM_030751.6.

Notably, the clinical heterogeneity observed in PPCD3 patients, ranging from remaining asymptomatic throughout life to requiring corneal transplantation during adolescence, may be associated with the diversity of mutations or deletions in the coding region of the *ZEB1* gene (Jang et al., 2014; Chung et al., 2017; Fernández-Gutiérrez et al., 2022). Studies have demonstrated that *ZEB1* (OMIM #189909), functioning as a transcriptional repressor, plays a pivotal role in epithelial-endothelial cell lineage transition and the development of neural crest-derived structures (particularly corneal endothelium) during embryogenesis (Evans et al., 2015). ZEB1 contains three core domains: a N-terminal zinc finger (NZF), central homeodomain (HD), and C-terminal zinc finger cluster (CZF), along with functional domains (SBD, CBD, CID) that mediate protein interactions. Through SMARCA4/BRG1 recruitment to its N-terminus, ZEB1 induces epithelial-mesenchymal transition (EMT) by repressing epithelial markers, such as E-cadherin (Schmalhofer et al., 2009). Mutations in *NZF* and other sites may affect the normal function of *ZEB1*. For example, a *ZEB1* mutation failed to suppress CDH1 in epithelium, and reduced expression of *ZEB1* may lead to insufficient binding to the E2 box, and thus repression of *COL4A3* in the corneal endothelium of patients with PPCD3 (Chung et al., 2016). In this study, we identified a heterozygous missense variant c.13C>G (p.P5A, rs753301298) in *ZEB1*’s exon 1/NZF domain, causing a Pro5Ala substitution (Figure 6). Computational analyses (Polyphen2, SIFT, MutationTaster, Fathmm-MKL, and CADD) (Table 2) and structural modeling suggested pathogenicity, supported by high evolutionary conservation (GERP++ = 4.12), low gnomAD frequency (PM2) and predicted structural perturbations. Moreover, according to the ACMG guidelines (Richards et al., 2015), the variant is likely to be a VUS, though its potential functional impact (PP3) correlates with our patients’ endothelial dystrophy. Notably, although *ZEB1* missense variants typically associate with KC/FECD (vs. truncating mutations in PPCD3), our findings and recent reports (Mazzotta et al., 2014; Bykhovskaya et al., 2016), suggest missense variants may also contribute to PPCD3 pathogenesis, particularly in cases of overlap of KC and PPCD3.

In addition to *ZEB1*, variations in the *ZNF469* gene have been implicated in keratoconus pathogenesis. The *ZNF469* gene (NM_001127464) encodes a poorly conserved C2H2 zinc finger protein with five exons. While previously unreported in PPCD3, *ZNF469* critically regulates central corneal thickness (CCT), and its variants predispose to keratoconus and brittle cornea syndrome type 1 (Abu et al., 2008; Loukovitis et al., 2018). Here, we identified a Mendelian-inherited heterozygous non-frameshift mutation (c.3093_3104del, p.D1035_K1038del) in *ZNF469* exon 3, co-occurring with the *ZEB1* rs753301298 variant in affected family members (Figure 3). This in-frame deletion causes the loss of four zinc finger domain residues (1035-1038) and protein conformational changes (Figure 4).

In humans, the corneal stroma accounts for 90% of the corneal thickness, and resident keratinocytes deposit a collagen-rich extracellular matrix. *ZNF469* mutations downregulate stromal ECM genes, including EGF-like repeat and discoidin I-like domain-containing protein 3 (EDIL3), collagen alpha-1 (IV) chain (COL4A1), collagen alpha-1 (XI) chain (COL11A1), transforming growth factor beta-2 (TGFb2) and hyaluronan and proteoglycan link protein 1 (HAPLN1) (Lechner et al., 2013). Our PPI network analysis also revealed interactions between *ZNF469* and CCT candidate genes, including *COL5A1*, *COL1A1,* and other genes that regulate eyeball development, such as *VSX1* (causing KC and corneal dystrophy) and *CHST14* (Ehlers-Danlos Syndrome candidate gene, Figure 5). Therefore, variants in *ZNF469* could induce abnormal corneal development through disturbing these pathways. The protein sequence of *ZNF469* shows 30% homology with the helical parts of *COL1A1*, *COL1A2* and *COL4A1*, all of which are abundantly expressed in the cornea (Abu et al., 2008). There is evidence to suggest that patients with KC may have a dysregulation of collagen homeostasis, because 70% of the components in the cornea are collagen, especially type I collagen (Critchfield et al., 1988; Kenney et al., 1997).

### 4.2 The pathogenic synergy between ZEB1 and ZNF469 in both PPCD3 and KC

Numerous cases have demonstrated a correlation between PPCD3 and KC within the same patient’s cornea (Zhang et al., 2021). The two conditions share similar pathological features, including corneal stromal thinning, extracellular matrix (ECM) remodeling, and inflammatory cell infiltration (Jeang et al., 2021; Santodomingo-Rubido et al., 2022), suggesting potential involvement of common underlying mechanisms. Previous studies have focused primarily on separate genetic analyses of PPCD3 or KC. For instance, Liyan Xu et al. identified eight hub genes (LAMB3, LAMA3, LAMA1, ITGA6, ITGA3, COL6A3, COL6A2, and COL6A1) as key candidate genes for KC (Ren et al., 2023), while Shaowei Li et al. proposed that eleven genes (CAT, COL12A1, FLG, HKDC1, HSPG2, PLOD1, ITGA2, TFAP2B, USH2A, WNT10A, and COL6A5) might be associated with KC pathogenesis in Chinese patients (Song et al., 2024). However, there have been few reports focusing on familial cases with PPCD3-KC overlapping symptoms and their associated genetic analyses. The present study identified pathogenic mutations in *ZEB1* and *ZNF469* in a pedigree exhibiting PPCD3-KC overlapping symptom (Figures 3, 4). Recently, studies have established that *ZEB1* (Lin K. et al., 2024) and *ZNF469* (Bao et al., 2023) are critically involved in ECM homeostasis. Furthermore, our protein-protein interaction (PPI) analysis revealed a close association between *ZEB1* and *HDAC1*. HDAC1 regulates ECM stability by deacetylating histones (e.g., H3K27ac), thereby suppressing the transcription of collagen genes (e.g., *COL1A1*, *COL3A1*) (Li et al., 2017). This mechanism mitigates fibrosis, as seen in corneal scarring, where HDAC1 upregulation reduces type I/III collagen deposition. Notably, *COL1A1* and *COL3A1* also exhibit significant interaction with *ZNF469* (Figure 5). These findings suggest that the co-mutation of *ZEB1* and *ZNF469* may cooperatively regulate ECM dyshomeostasis in PPCD3-KC overlapping symptoms, potentially through disrupting HDAC1-mediated transcriptional dysregulation of collagen genes (e.g., *COL1A1* and *COL3A1*).

In addition, the involvement of ZEB1 in ocular inflammatory processes remains poorly characterized. Li et al. demonstrated that ZEB1, in conjunction with CREB, binds to promoters of pro-inflammatory cytokines IL-1β and IFN-γ, upregulating their expression in corneal epithelial cells via p38 MAPK signaling (Li et al., 2010). This finding establishes ZEB1 and CREB as critical regulators in immune-mediated ocular surface squamous metaplasia. Complementing this, Park et al. showed that Epstein-Barr virus infection activates both Snail and ZEB1, promoting their nuclear translocation, a process that may drive epithelial-mesenchymal transition through loss of epithelial characteristics and acquisition of mesenchymal traits (Park et al., 2014). However, studies investigating the potential role of *ZNF469* in corneal inflammation remain scarce. Consequently, whether these two genes share common mechanisms in mediating inflammatory responses in both PPCD3 and keratoconus warrants further investigation.

### 4.3 Clinical translation value and significance of our genetic findings

The largest published cohort study to date identified *ZEB1* mutations in 25% of PPCD cases (8/32 probands) (Aldave et al., 2007), with reported detection rates varying significantly across studies (9.1%–45.4%) (Aldave et al., 2007; Dudakova et al., 2019). Notably, Zhou et al. demonstrated that only 1.6% of patients with keratoconus (KC) harbor *ZNF469* mutations (Lin Q. et al., 2024). These findings collectively underscore the limited phenotypic contribution of single-gene mutations. In our pedigree, 100% of family members (3/3 probands) carrying dual *ZEB1* and *ZNF469* mutations exhibited PPCD3-KC overlapping phenotypes (Table 1). Although the generalizability of single-family studies remains constrained, this striking association strongly implicates *ZEB1*-*ZNF469* digenic interactions in driving the PPCD3-KC phenotypic spectrum. For clinical management and preoperative evaluation of refractive surgery candidates in this cohort, meticulous consideration must be given to their elevated risk of developing PPCD3 or KC postoperatively, warranting risk-stratified surveillance protocols and tailored pre-/intraoperative prophylactic interventions.

Two novel variants in ZEB1 and ZNF469 were identified in this study as genetic factors associated with KC and PPCD. This finding highlights the importance of screening for ZEB1 and ZNF469 in patients who are considering refractive surgery and have a family history of KC and PPCD, as well as those exhibiting high corneal astigmatism or irregular corneal morphology. Such screening could facilitate preoperative risk assessment for refractive surgeries. For individuals with subtle corneal abnormalities but harboring mutations in ZEB1 or ZNF469, targeted dynamic monitoring is recommended (e.g., gene-specific matrix) (Henderson et al., 2024). We can develop personalized testing protocols based on the results of genetic screening. Additionally, implementing multimodal intervention strategies has been shown to significantly reduce the incidence of KC and PPCD following refractive surgery. These strategies may include combined corneal cross-linking (Santodomingo-Rubido et al., 2022), genetic counseling, and interdisciplinary management.

## 5 Conclusion

In conclusion, this study demonstrated the importance of a thorough ocular examination, especially the cornea, and a gene screening before cornea refractive surgery. Genetic abnormalities would increase the risk of post refractive complications, such as KC. Two novel variants in *ZEB1* and *ZNF469* were identified in this study as genetic factors associated with KC and PPCD. With the application of advanced genetic analyses in the clinic, ocular hereditary disorders such as KC or PPCD can be detected and diagnosed very early before clinical onset to avoid the risk of cornea refractive surgery.

## Data Availability

The datasets presented in this study can be found in online repositories. The names of the repository/repositories and accession number(s) can be found below: https://www.ncbi.nlm.nih.gov/, SCV003842308, SCV003842309, SCV003842310, SCV003842311 and SCV003842314.

## Supplementary material

SUPPLEMENTARY FIGURE S1 The corneal topography (Pentacam) reports for family member III.1(proband). 1a: Pentacam preoperative. The means of posterior elevation of the cornea (PEC) were 9 µm (right eye) and 14 µm (left eye). The central corneal thicknesses (CCT) were 519 and 528 µm in the right and left eye, respectively. 1b: Pentacam 6 months postoperative. The means of posterior elevation of the cornea (PEC) were 6 µm (right eye) and 29 µm (left eye). The central corneal thicknesses (CCT) were 433 and 455 µm in the right and left eye, respectively. 1c: Pentacam 1 year postoperative. The means of posterior elevation of the cornea (PEC) were 19 µm (right eye) and 44 µm (left eye). The central corneal thicknesses (CCT) were 423 and 445 µm in the right and left eye, respectively. 1d: Pentacam 1.5 years postoperative. The means of posterior elevation of the cornea (PEC) were 21 µm (right eye) and 45 µm (left eye). The central corneal thicknesses (CCT) were 426 and 445 µm in the right and left eye, respectively.

SUPPLEMENTARY FIGURE S2 The corneal topography (Pentacam) reports for family member III.2. 2a: Pentacam preoperative. The means of posterior elevation of the cornea (PEC) were 14 µm (right eye) and 13 µm (left eye). The central corneal thicknesses (CCT) were 564 and 567 µm in the right and left eye, respectively. 2b: Pentacam 1 month postoperative. The means of posterior elevation of the cornea (PEC) were 18 µm (right eye) and 15 µm (left eye). The central corneal thicknesses (CCT) were 454 and 449 µm in the right and left eye, respectively. 2c: Pentacam 6 months postoperative. The means of posterior elevation of the cornea (PEC) were 26 µm (right eye) and 30 µm (left eye). The central corneal thicknesses (CCT) were 465 and 461 µm in the right and left eye, respectively.

SUPPLEMENTARY FIGURE S3 The corneal topography (Pentacam) reports for family member II.2 The means of posterior elevation of the cornea (PEC) were 12 µm (right eye) and 9 µm (left eye). The central corneal thicknesses (CCT) were 532 and 528 µm in the right and left eye, respectively.

## Abbreviations

LASIK, laser-assisted *in situ* keratomileusis; PRK, photorefractive keratectomy; SMILE, small-incision lenticule extraction; KC, keratoconus; PPCD, posterior polymorphous corneal dystrophy; PPCD3, posterior polymorphous corneal dystrophy subtype 3; BCVA, best-corrected visual acuity; OCT, optical coherence tomography; ECD, endothelium cell density; ES, exome sequencing; PCR, polymerase chain reaction; PPI, protein-protein interaction; UDVAs, uncorrected distance visual acuities; PEC, posterior elevation of the cornea; VUS, variant of uncertain significance; HGMD, Human Gene Variant Database; NZF, amino-terminal zinc finger; HD, homeodomain; SBD, SMAD binding domain; CBD, CAF binding domain; CID, CtBP interacting domain; EMT, Epithelial-mesenchymal transition; FECD, Fuchs endothelial corneal dystrophy; CCT, central corneal thinning; BCS, brittle cornea syndrome; EDIL3, EGF-like repeat and discoidin I-like domain-containing protein 3; TGFb2, transforming growth factor beta-2; HAPLN1, hyaluronan and proteoglycan link protein 1; IVCM, *in vivo* confocal microscopy.
