# Supplementary figures and images for: Polymorphous corneal dystrophy subtype 3 and keratoconus aggravation after corneal refractive surgery in a three-generation family carrying both ZEB1 and ZNF469 pathogenic variant
Source: Front Genet. 2025 Jun 6;16:1603019. doi: 10.3389/fgene.2025.1603019 (PMC12179129; doi:10.3389/fgene.2025.1603019)

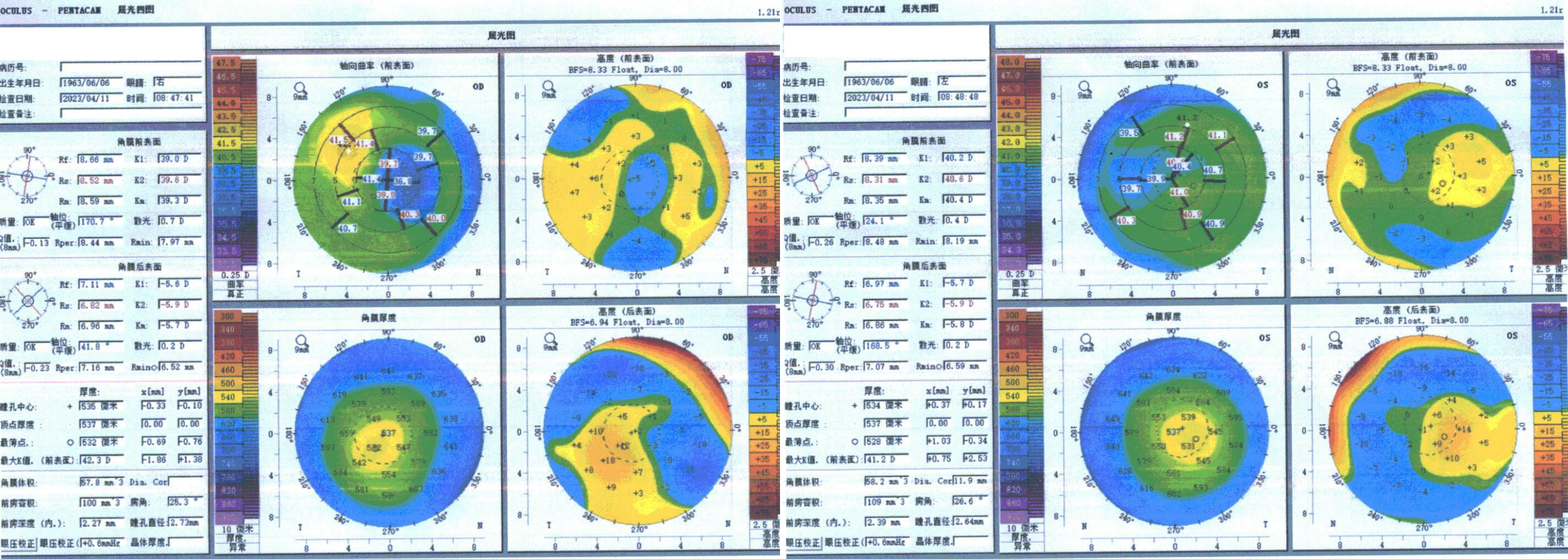

Supplement: Supplementary file 1 [file Image3.tif]

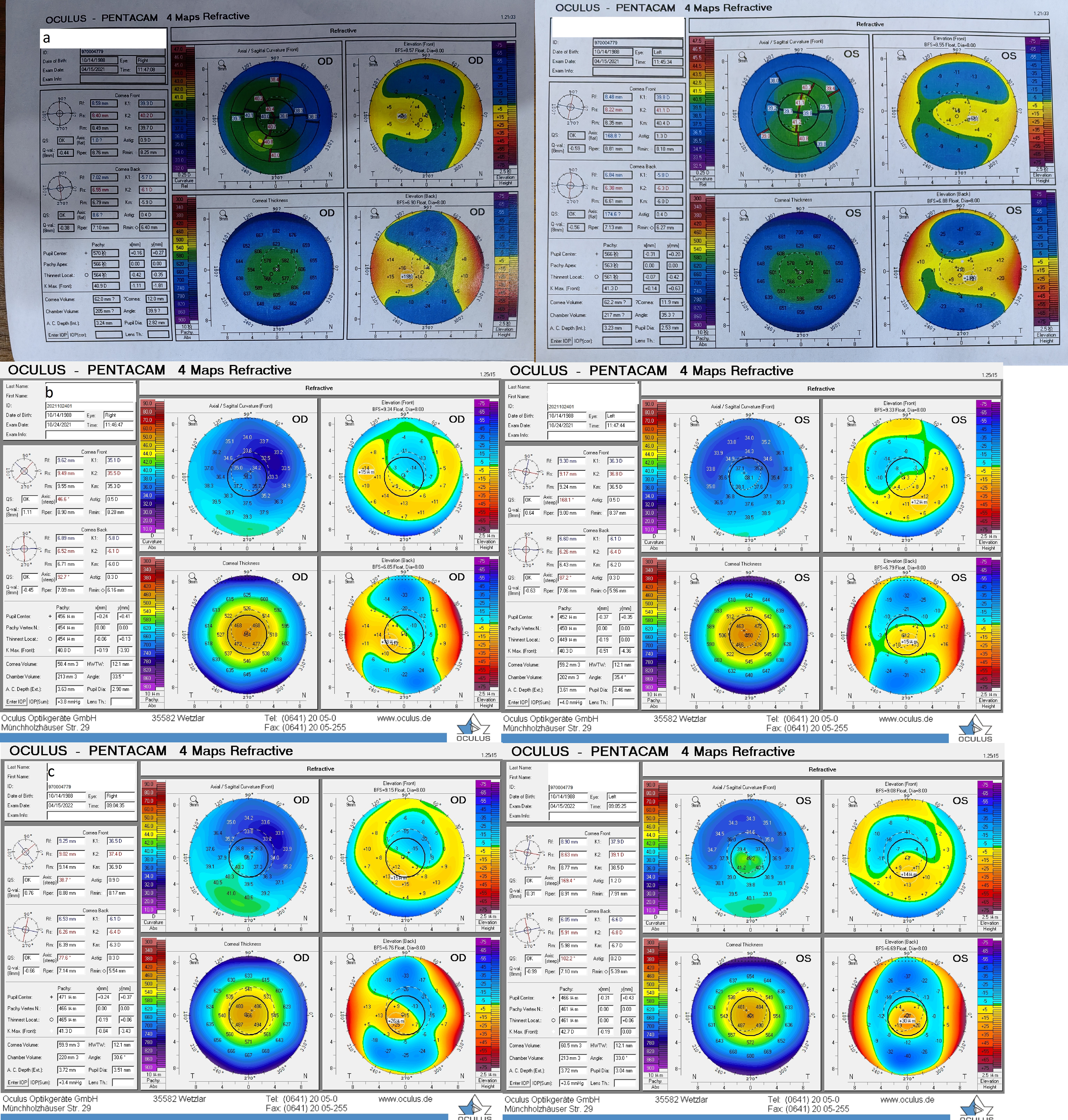

Supplement: Supplementary file 2 [file Image2.tif]

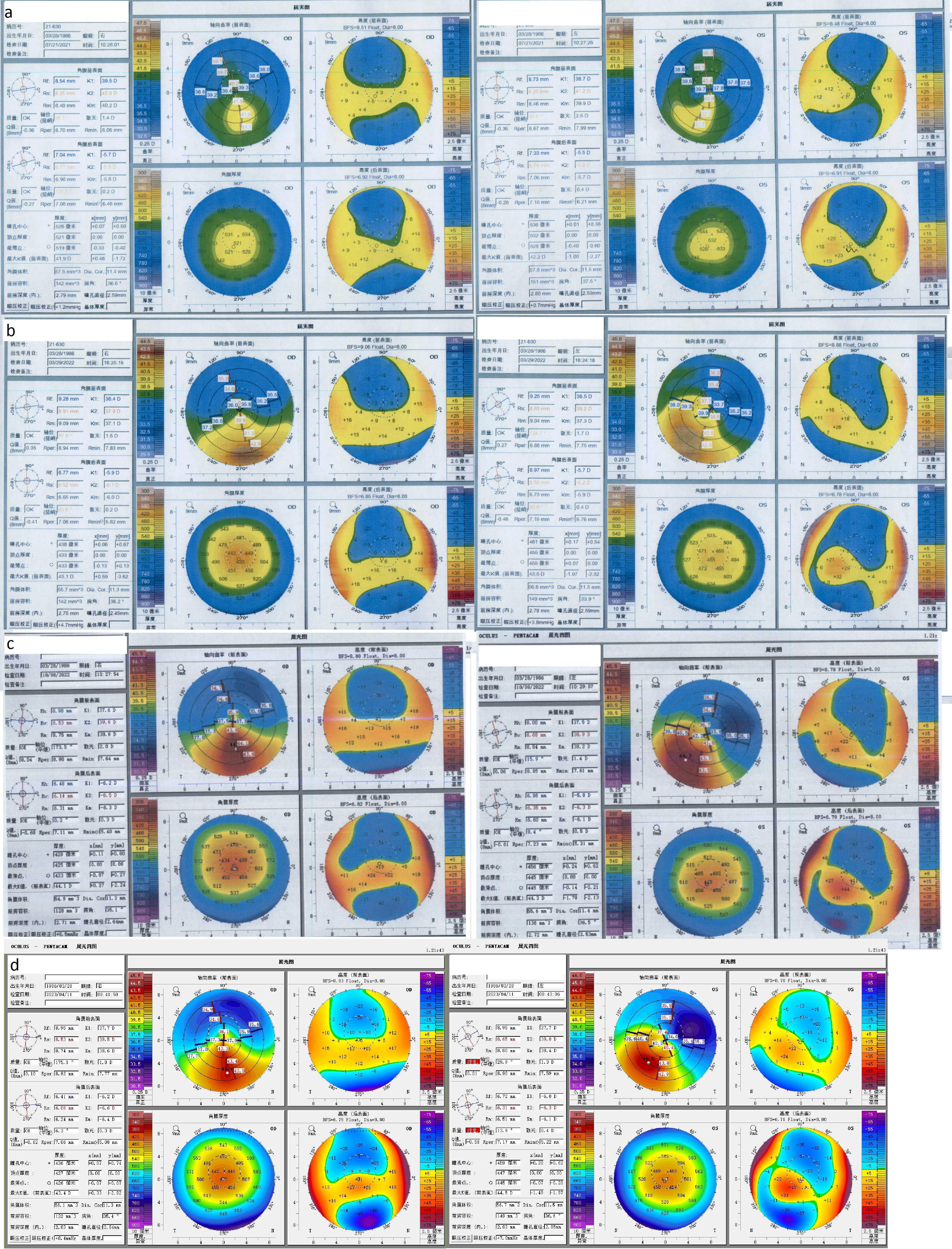

Supplement: Supplementary file 3 [file Image1.tif]
